# Improving Suicidal Ideation Detection in Social Media Posts: Topic Modeling and Synthetic Data Augmentation Approach

**DOI:** 10.2196/63272

**Published:** 2025-06-11

**Authors:** Hamideh Ghanadian, Isar Nejadgholi, Hussein Al Osman

**Affiliations:** 1 School of Electrical Engineering and Computer Science University of Ottawa Ottawa, ON Canada; 2 National Research Council Canada Ottawa, ON Canada

**Keywords:** suicidal ideation detection, large language models, synthetic data, topic modeling, social media

## Abstract

**Background:**

In an era dominated by social media conversations, it is pivotal to comprehend how suicide, a critical public health issue, is discussed online. Discussions around suicide often highlight a range of topics, such as mental health challenges, relationship conflicts, and financial distress. However, certain sensitive issues, like those affecting marginalized communities, may be underrepresented in these discussions. This underrepresentation is a critical issue to investigate because it is mainly associated with underserved demographics (eg, racial and sexual minorities), and models trained on such data will underperform on such topics.

**Objective:**

The objective of this study was to bridge the gap between established psychology literature on suicidal ideation and social media data by analyzing the topics discussed online. Additionally, by generating synthetic data, we aimed to ensure that datasets used for training classifiers have high coverage of critical risk factors to address and adequately represent underrepresented or misrepresented topics. This approach enhances both the quality and diversity of the data used for detecting suicidal ideation in social media conversations.

**Methods:**

We first performed unsupervised topic modeling to analyze suicide-related data from social media and identify the most frequently discussed topics within the dataset. Next, we conducted a scoping review of established psychology literature to identify core risk factors associated with suicide. Using these identified risk factors, we then performed guided topic modeling on the social media dataset to evaluate the presence and coverage of these factors. After identifying topic biases and gaps in the dataset, we explored the use of generative large language models to create topic-diverse synthetic data for augmentation. Finally, the synthetic dataset was evaluated for readability, complexity, topic diversity, and utility in training machine learning classifiers compared to real-world datasets.

**Results:**

Our study found that several critical suicide-related topics, particularly those concerning marginalized communities and racism, were significantly underrepresented in the real-world social media data. The introduction of synthetic data, generated using GPT-3.5 Turbo, and the augmented dataset improved topic diversity. The synthetic dataset showed levels of readability and complexity comparable to those of real data. Furthermore, the incorporation of the augmented dataset in fine-tuning classifiers enhanced their ability to detect suicidal ideation, with the *F*_1_-score improving from 0.87 to 0.91 on the University of Maryland Reddit Suicidality Dataset test subset and from 0.70 to 0.90 on the synthetic test subset, demonstrating its utility in improving model accuracy for suicidal narrative detection.

**Conclusions:**

Our results demonstrate that synthetic datasets can be useful to obtain an enriched understanding of online suicide discussions as well as build more accurate machine learning models for suicidal narrative detection on social media.

## Introduction

Suicide is a pressing global public health issue with far-reaching consequences for individuals, families, and communities. The World Health Organization [1] has highlighted the alarming increase in suicide rates in recent years, emphasizing the urgent need for effective prevention strategies [2]. Traditionally, identifying individuals at risk of suicide has relied on clinical assessments and crisis hotlines. However, the widespread adoption of social media has opened new avenues for early detection and intervention [3].

Natural language processing (NLP) techniques have emerged as a promising tool for suicide detection, leveraging machine learning to analyze textual data shared on social media platforms [4-6]. Social media datasets have become invaluable in the study of suicidal ideation. As more people express their thoughts and emotions online, these datasets offer a unique glimpse into individuals’ digital lives, providing rich data for research and mental health support [7]. Researchers use these datasets to analyze text, identifying patterns and linguistic cues associated with suicidal ideation [8-10].

However, while social media provides a wealth of information, it is not without its limitations. For instance, social media platforms tend to attract a younger demographic, which may skew the data and make them less representative of older populations. Additionally, the open nature of social media may encourage users to share certain aspects of their experiences while discouraging the disclosure of more personal or sensitive factors [11]. These biases can have significant implications for machine learning models trained on social media data. If the data are not representative of all demographics or fail to capture less frequently discussed factors, the models may struggle to generalize effectively. For example, a model trained on data that overrepresent certain themes or demographics may perform poorly when encountering posts from underrepresented groups or those addressing less common topics. This could lead to false negatives, where the model fails to identify at-risk individuals, or biased outputs, where the model perpetuates stereotypes or stigmatization present in the training data.

To address these limitations, it is crucial to complement social media datasets with information from other reliable sources. Within psychology, extensive research has been conducted to understand the underlying psychological, social, and environmental factors contributing to suicidal thoughts and behaviors. Several investigations have explored how psychological factors, including depression, anxiety, a sense of hopelessness, and feelings of low self-worth, influence the emergence of suicidal thoughts [12-14]. These studies have investigated the strong association between suicidal thoughts and conditions like depression [15,16], bipolar disorder [17], borderline personality disorder [18], and substance abuse [19,20]. The studies used a combination of quantitative and qualitative methods to explore the factors contributing to suicide [21-24].

Our objective in this study is to identify gaps between the risk factors represented in social media data and those established in psychology literature. We hypothesize that social media data do not fully capture empirically established factors related to suicidal ideation, particularly those that may be underrepresented due to demographic or disclosure biases. To examine this, we conducted a comprehensive analysis of suicide-related social media datasets using topic modeling techniques to identify themes and topics [25]. We then compared the extracted themes with factors outlined in psychology literature to locate any significant gaps. When such gaps were identified, we explored the potential of augmenting real-world data with synthetic data generated by advanced language models like GPT-3.5 Turbo to improve the representation of underreported factors. The use of synthetic data has gained traction in recent years, particularly with the advent of generative models that can produce high-quality, realistic data [26,27]. This study presents a novel approach for identifying and mitigating topic gaps in suicidal ideation detection models, aiming to improve their generalizability and fairness.

## Methods

### Study Methodology

**Figure 1:**
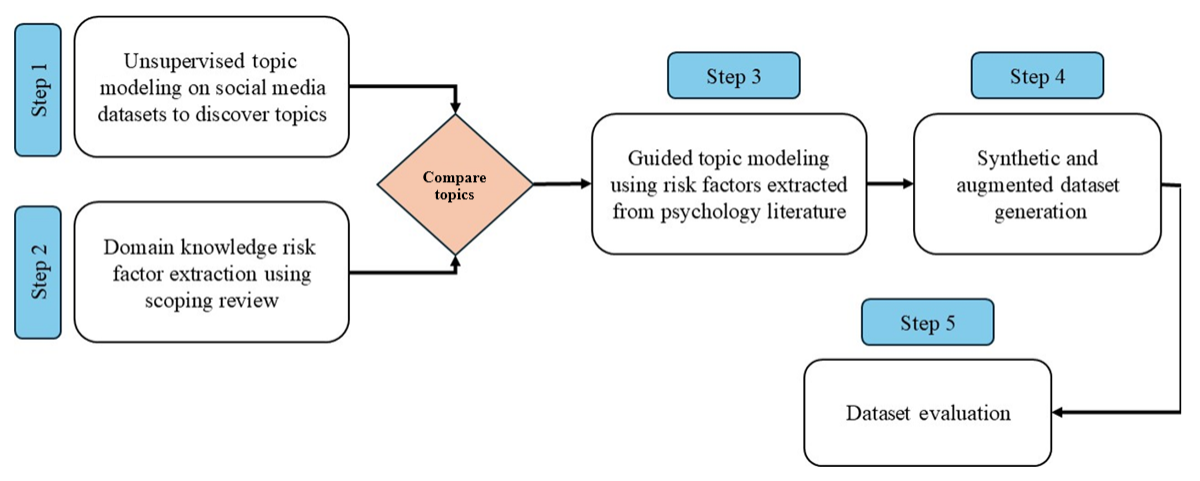
Overview of the multistep methodology used in this study to assess topic gaps in suicidal ideation detection from Reddit social media posts.

In this subsection, we elaborate on our study methodology. Figure 1 illustrates how the study methodology was developed. Our approach included the following steps:

- Step 1 (unsupervised topic modeling): We performed unsupervised topic modeling to discover the topics and risk factors mostly discussed on social media.
- Step 2 (domain knowledge extraction): We extracted relevant social risk factors from psychology literature to ground the content assessment of datasets in these factors.
- Step 3 (guided topic modeling): We compared topics extracted from social media and the literature. We shifted toward supervised guided topic modeling on social media using discovered topics from the literature to identify underrepresented and missing risk factors.
- Step 4 (synthetic and augmented dataset): We extended and annotated the existing synthetic datasets by incorporating additional risk factors that were missing from existing datasets. Moreover, we created an augmented dataset by combining synthetic data with real social media data to enhance its generalizability and topic coverage.
- Step 5 (dataset evaluation): To evaluate the utility of synthetic data, we analyzed both real and synthetic datasets by assessing the complexity, readability, and diversity of the text within each dataset. Additionally, we trained state-of-the-art classifiers using real-world, synthetic, and augmented datasets. Subsequently, the performance of these classifiers was evaluated by testing them on both real-world and synthetic test sets.

### Unsupervised BERTopic

In this subsection, we present the methodology for conducting topic modeling using BERTopic [28], a state-of-the-art deep learning approach for discovering topics in text data. Our objective was to assess the quality, diversity, and coverage of datasets collected from social media in relation to suicidal ideation.

BERTopic leverages transformer-based language models as embedding models, combined with clustering methods for topic extraction [29]. BERTopic integrates transformer-based techniques with term frequency–inverse document frequency (TF-IDF) to create compact, interpretable clusters, preserving the most relevant terms in topic descriptions. This approach leverages deep learning and is mostly used with the sentence transformers embedding model, which supports document embedding extraction in more than 50 languages [30]. BERTopic’s topic modeling procedure involves 3 main stages: document embeddings, document clustering, and topic representation.

#### Text Embedding

Text embedding refers to the vector representation of text within a multidimensional space where textual contents conveying similar meanings exhibit similar embeddings. In this project, we used the “*SentenceTransformers*” Python framework for state-of-the-art sentence and text embeddings [31]. Although there are many models for text embeddings, we used the sentence transformers “*all-MiniLM-L6-v2*” model as it has been shown to be one of the best-performing models within the BERTopic framework [32].

#### Topic Extraction

In this step, similar documents or sentences are grouped based on their content. It is a method of organizing large amounts of textual information into meaningful categories or clusters, providing a high-level overview of the information contained within. Before clustering the embeddings, a dimensionality reduction is implemented, as embeddings are often high in dimensionality. In this work, we used the UMAP (Uniform Manifold Approximation and Projection) algorithm to reduce the dimensionality of the embeddings because it can capture both the local and global structures of high-dimensional data in lower-dimensional space [33]. It has also been proven that for short text clustering, UMAP demonstrates superior results [34,35]. The hyperparameter space involved in UMAP is manually inspected, and based on the performance of the model and presented topics, the best parameters are selected. The number of neighbors, the number of components, and the minimum distance of each component were selected as 15, 5, and 0, respectively.

Following the dimensionality reduction of our input embeddings, we needed to cluster them into groups of similar embeddings to extract our topics. The method used in this paper is HDBSCAN (Hierarchical Density-Based Spatial Clustering of Applications with Noise), introduced by Campello et al [36]. This method is based on the density clustering method that finds clusters of different shapes and identifies outliers where possible. Similar to UMAP, the parameters of this model are manually inspected, and the proper parameters are chosen. There is no automated method for determining values for HDBSCAN. The parameters should be set manually based on domain knowledge and understanding of the dataset. Hence, we selected 10 and 5 as the minimum cluster size and sample number in each cluster, respectively.

#### Keyword Extraction for Topics

In this step, class-based term frequency–inverse document frequency (C-TF-IDF) scores are used to identify a set of keywords that represent the topic for a better interpretation of the topic’s content. TF-IDF is a technique used to extract features from text documents, which is achieved by combining 2 components: term frequency and inverse document frequency. Term frequency represents the simple word count within a document, treating each word count as a feature, and it is calculated by dividing the number of times the term occurs in the document by the total number of terms in the document. Inverse document frequency gauges the informativeness of specific words by measuring their frequency within a document relative to their frequency across all other documents. C-TF-IDF is similar to TF-IDF but is adopted for multiple classes by joining all documents per class. Thus, each class is converted to a single document instead of a set of documents. After calculating the C-TF-IDF scores for all words in each topic, words with the highest scores are chosen as keywords associated with that topic.

### Social and Psychological Knowledge Extraction

We conducted a scoping review to offer a current and thorough synthesis of psychology studies, aiming to identify topics, themes, and risk factors related to the sensitive and complex issue of suicidal ideation. Scoping reviews serve as a method to assess the scope of literature on a particular subject, providing insights into the available research and offering a broad overview of its focus [37]. Additionally, they highlight the types of evidence that guide practice in the field and examine how the research has been conducted [38]. Our protocol was developed using the scoping review methodological framework proposed by Arksey and O’Malley [39] and further refined by Peters et al [40].

#### Research Question

The central research question guiding this review was as follows: “What are the most frequently reported risk factors associated with suicidal ideation in the psychology and mental health literature?” By answering this question, the review seeks to provide a clearer understanding of the key factors that contribute to the emergence of suicidal thoughts, ultimately informing future research and interventions aimed at preventing suicide and supporting individuals at risk.

#### Search Strategy

We scoured prominent academic databases, such as PubMed, PsycArticles, ScienceDirect, and Google Scholar, employing a systematic approach. Our search strategy involved using identical keywords across all databases: “suicidal ideation” and “suicide risk factors.”

#### Eligibility Criteria

To be eligible for inclusion, studies were required to be peer-reviewed review or systematic review publications that investigated risk factors associated with suicidal ideation. Eligible studies had to be published between January 2014 and August 2024, address all forms of suicidal behavior including ideation and attempts, and focus on the general population.

Studies were excluded if they focused on specific groups that could introduce additional factors into the analysis, were published in a language other than English, or were not peer-reviewed publications, such as book chapters.

#### Study Selection

All identified publications were initially screened for relevance based on abstract and title. Subsequently, the full text of selected publications was assessed for eligibility. Furthermore, the reference lists of eligible papers were used to identify additional studies.

This exploration allowed us to identify a wide array of relevant review papers, forming the foundation of our research. Subsequently, we reported the most common topics among all the selected research articles. Based on our analysis of the literature, several social and psychological factors were consistently reported in relation to suicidal ideation in psychology. These topics have not been listed in a specific order of importance but represent the consistently reported themes in the literature reviewed.

#### Mental Health Disorders and Personality Traits

“Depression” is a well-documented and significant risk factor for suicide. The persistent feelings of sadness and emotional pain that characterize depression can lead individuals to contemplate or attempt suicide as a means of escape from their suffering. It is vital to recognize the signs of depression, offer support, and connect individuals to mental health professionals and resources for effective treatment and intervention [41-43].

“Anxiety” disorders are commonly associated with suicidal ideation. The chronic emotional distress and physical symptoms associated with severe anxiety can contribute to the development of suicidal thoughts [44].

“Posttraumatic stress disorder” (PTSD) is strongly associated with an increased risk of suicidal ideation. Individuals who have experienced traumatic events may struggle with the emotional aftermath, including intrusive memories, hyperarousal, and avoidance of reminders, which can lead to constant emotional distress and a sense of being overwhelmed. These constant feelings of traumatic memories can contribute to thoughts of suicide [45,46].

“Bipolar disorder,” formerly called manic depression, is a mental health condition that causes extreme mood swings. These include emotional highs (also known as mania) and lows (also known as depression) [47]. Suicide attempts and completed suicide are significantly more common in patients with bipolar disorder when compared with the general population [47,48].

“Schizophrenia” is a severe mental disorder that disrupts both cognitive and social functioning, often resulting in the onset of additional health conditions [49]. Schizophrenia is strongly linked to an increased risk of suicidal ideation. The distress caused by its symptoms, feelings of isolation, and perceived loss of control over one’s mind can contribute to a deep sense of hopelessness. Additionally, the stigma and social withdrawal often associated with schizophrenia may exacerbate these feelings, further increasing the risk of suicide [50,51].

“Borderline personality disorder” is a prevalent mental health condition linked to elevated suicide rates, significant functional impairment, frequent co-occurrence with other mental disorders, and extensive treatment needs. Long-term outcome studies of patients with borderline personality disorder have documented a high rate of suicide completion [52,53].

The expression and experience of “anger” have been reported as influential factors in suicidal ideation. Unresolved anger and intense emotional turmoil can drive individuals toward suicidal thoughts and actions. Anger, when left unmanaged, can escalate distress and lead to impulsive decisions with dire consequences. Recognizing and managing anger are crucial facets of suicide detection [41,54].

“Perfectionism,” marked by excessively high standards and self-criticism, has been identified as a psychological factor related to suicidal ideation. The relentless pursuit of unattainable standards can lead to feelings of inadequacy and despair, increasing the risk of suicidal ideation. Recognizing the need for balance and self-compassion is pivotal in addressing this risk factor [41].

Feelings of “hopelessness,” characterized by a pervasive sense of despair and an inability to envision a better future, can be a potent predictor of suicidal behavior. Those burdened by overwhelming hopelessness may see suicide as the only means of escape from their emotional suffering [41,42,55].

#### Substance Abuse

Alcohol and drug misuse is a significant risk factor for suicidal ideation and behavior [56]. The connection between substance abuse and suicide is complex, as substances like drugs and alcohol can impair judgment and exacerbate underlying emotional distress. Individuals who struggle with addiction may turn to substances as a means of coping with psychological pain, and when combined with impaired decision-making, this can increase the likelihood of suicidal thoughts and actions [57-59].

#### Sociodemographic Status

Prolonged “unemployment” can erode self-esteem, create financial difficulties, and contribute to feelings of hopelessness, increasing the risk of suicide. Government support and job assistance programs can help mitigate this risk [55]. “Financial hardships” can trigger intense stress, making individuals vulnerable to suicide. Economic support and resources are instrumental in addressing this risk factor [44,60].

“Education pressure,” including exams and expectations, can lead to emotional turmoil and an increased risk of suicidal ideation, particularly among students. Educational institutions must provide resources for coping with academic stress [61,62].

“Sexual minority” individuals, such as those who identify as lesbian, gay, bisexual, transgender, queer (LGBTQ+), often face unique stressors related to their sexual orientation or gender identity [63]. Discrimination, prejudice, and stigma can lead to feelings of isolation, rejection, and psychological distress. Research consistently shows that sexual minority individuals are at a higher risk for suicidal ideation and attempts compared to their heterosexual counterparts [64-67].

#### Abuse

“Bullying,” including physical, verbal, or cyber bullying, has consistently emerged as a significant topic related to suicidal ideation. The experience of bullying can lead to social isolation, low self-esteem, and emotional distress, contributing to the development of suicidal thoughts [44,68-70].

“Sexual abuse” is a recognized risk factor for suicide, which includes any sexual activity that occurs without consent, also referred to as sexual assault or sexual violence [71]. Dissociation is a common response to sexual abuse, and higher levels of dissociation have been associated with self-harm, suicidal thoughts, and suicide attempts [72,73].

#### Family-Related Issues

“Family-related stressors,” such as conflict, dysfunctional dynamics, and poor communication, can significantly impact an individual’s emotional well-being and contribute to suicidal thoughts. Strengthening family relationships and providing support to those affected are essential in mitigating this risk factor [43,55].

Difficulties in “relationships,” including conflicts, breakups, and marital dissatisfaction, have been reported as significant topics in relation to suicidal ideation. Relationship problems can contribute to emotional distress and feelings of hopelessness, leading to thoughts of suicide [55].

The death of family members or friends has been reported as a risk factor associated with suicidal ideation. Grief, feelings of loneliness, and a sense of being unable to cope with the loss can increase the risk of suicidal thoughts [74].

#### Racism

Studies have consistently highlighted the significant impact of racial discrimination on suicidal ideation. Experiencing racism and racial prejudice can increase the risk of suicidal thoughts [75-77].

#### Immigration

The process of immigration, with its cultural adjustments, isolation, and uncertainty, can intensify stress and emotional distress, increasing the risk of suicide among immigrants. Providing support and resources tailored to the immigrant experience is essential [78,79].

#### Dementia

Dementia, particularly in its advanced stages, can lead to significant cognitive and emotional challenges. Individuals with dementia may experience confusion, memory loss, and personality changes, which can be distressing for both them and their caregivers. The experience of losing one’s cognitive abilities and identity can contribute to feelings of hopelessness and despair, leading to thoughts of suicide [80-82].

#### Chronic Physical Problems

Living with chronic physical health conditions can be emotionally taxing, and individuals facing such challenges may be more susceptible to suicidal ideation. Chronic pain, disability, and limitations in physical functioning can erode one’s quality of life and lead to a sense of hopelessness [83]. Coping with the constant demands of managing a chronic condition can also contribute to emotional distress [84,85].

### Guided BERTopic

In this research, we began by applying unsupervised BERTopic to the social media datasets to identify key topics discussed within them. Next, we conducted a scoping review to examine established suicidal risk factors. We then compared the topics from the social media analysis with risk factors extracted from psychology literature. During this process, we observed that the effectiveness of topic modeling techniques in capturing domain-specific terms may be limited. To address this, we used guided topic modeling to ensure that certain specialized terms are detected and appropriately represented in the topics.

Guided topic modeling (supervised topic modeling) is an extension of traditional topic modeling that incorporates external information or guidance to influence the topic discovery process. In this project, we employed guided BERTopic [86], which introduces external information in the form of seed words to guide the algorithm in discovering topics that align with the specified guidance. This guidance helps to improve the relevance and coherence of the identified topics. Here, we used discovered suicidal risk factors from psychology literature in the Social and Psychological Knowledge Extraction section as categories to guide the topic modeling process. Each category comes with sets of seed words that are suggested using “Meriam-Webster Dictionary” [87] as the most related keywords for each category. This deliberate approach allowed us to align the resulting topics with known psychological factors that contribute to suicide risk, creating a more focused and domain-informed representation.

**Figure 2:**
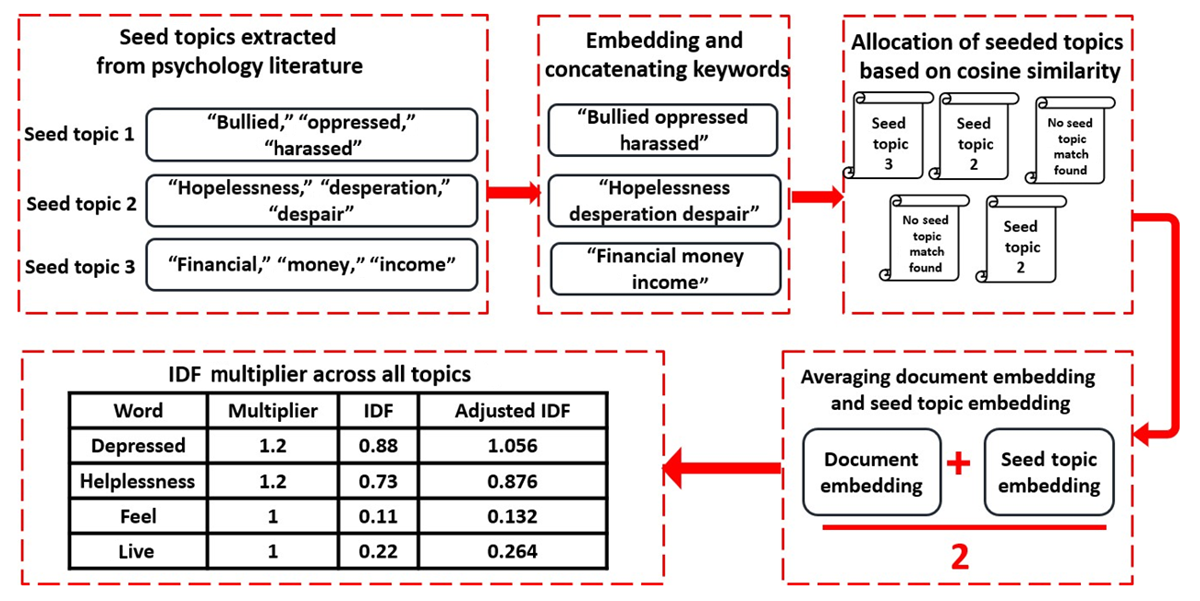
Architecture of guided BERTopic using seed terms derived from psychology literature. Illustration of how seed words based on established suicide risk factors from the literature were incorporated into the guided BERTopic algorithm to steer topic discovery in Reddit social media posts for suicidal ideation detection. IDF: inverse document frequency.

Guided BERTopic involves 2 primary steps. First, the BERTopic algorithm generates embeddings for each seeded topic by concatenating them and passing them through the document embedder. As shown in Figure 2, these embeddings are compared to the existing document embeddings using cosine similarity and are then assigned a label. If a document is most similar to a seeded topic, it receives that topic’s label; otherwise, if it is most similar to the average document embedding, it will be categorized as an outlier (–1 label). UMAP then applies these labels in a semisupervised manner, guiding the dimensionality reduction process to emphasize the distinctions between seeded topics and potentially identify outliers, thereby steering the topic creation more effectively toward the seeded topics. Second, all words in the seed topic list are assigned to a multiplier with a value greater than 1. These multipliers are applied to increase the inverse document frequency values of the words across all topics by boosting the likelihood of a seeded topic word appearing in a topic. After having generated our topics using C-TF-IDF, we employed the *KeyBERTInspired* extraction technique that leverages BERT embeddings and simple cosine similarity to find keywords and key phrases that are most similar to a document. Then, we selected keywords with the highest C-TF-IDF score as representative seed topics for the guided topic modeling.

### Synthetic Data Generation

Synthetic data generation provides a practical solution for addressing data availability limitations by creating artificially generated data that closely resemble real-world data. Ghanadian et al [26] employed 3 generative large language models, namely GPT-3.5 Turbo, Flan-T5, and Llama2, to generate a synthetic dataset related to suicide. Their findings indicated that GPT-3.5 Turbo outperformed other generative models in creating synthetic data, that is, a classifier trained on a dataset generated by GPT-3.5 Turbo achieved higher *F*-scores when tested on the real-world and synthetic test sets. In our research, we used the dataset presented in the study by Ghanadian et al [26], along with domain knowledge–based risk factors we extracted, to generate a new dataset using GPT-3.5 Turbo. Moreover, we augmented 30% of the real dataset with a new synthetic dataset to report the practicality of the generated data for suicidal ideation detection.

### Dataset Analysis

For textual datasets, assessing the diversity of semantics and the sentence structure of textual content is crucial, even more so when dealing with synthetic datasets. To measure these qualities, we used 3 sets of metrics: complexity, readability, and entropy. Complexity refers to the intricacy and sophistication of the language used in a text, encompassing factors such as sentence structure, vocabulary richness, and syntactic intricacies. Readability pertains to the ease with which a text can be comprehended by its intended audience, considering elements such as sentence length, word difficulty, and overall coherence. Understanding complexity and readability in synthetic datasets aids in ensuring that the generated text aligns with linguistic patterns observed in real-world data. Moreover, these parameters facilitate an assessment of the synthetic dataset, specifically regarding the incorporation of suitable language complexities. This evaluation allows us to examine if synthetic data replicate language patterns akin to those found in genuine, human-generated content. Furthermore, entropy is a metric that measures the unpredictability of a text. It assesses the information content or disorder present in the dataset. In simpler terms, entropy in text datasets gauges how diverse or varied the words or characters are within the dataset. High entropy indicates a higher degree of unpredictability, suggesting a wider range of different words or characters used in the text. Conversely, low entropy implies a more predictable or ordered text with fewer variations in the words or characters used.

To measure the readability of text, we used the Flesch reading ease test [88], which quantifies readability based on sentence length and the number of syllables per word. As a benchmark, a high score indicates that the text is easily understood by an average 11-year-old, while a low score indicates that the text is best understood by university graduates. Table 1 presents the definition criteria for the Flesch reading ease test score.

**Table 1:** Flesch reading ease test score definition criteria.

Score	Difficulty
90-100	Very easy
80-89	Easy
70-79	Fairly easy
60-69	Standard
50-59	Fairly difficult
30-49	Difficult
0-29	Very confusing

Additionally, we used the type-token ratio [89] to assess the complexity of a text using lexical diversity measures. The basic idea behind that measure is that if the text is more complex, the author uses a more varied vocabulary, so there is a larger number of unique words [90].

Another selected metric was Shannon entropy [91], which is calculated based on the frequency of occurrence of different characters, words, or other linguistic units within the text. In word-based analysis, higher entropy suggests a wider range of vocabulary, showing greater linguistic diversity [92]. Calculation of Shannon entropy involves summing the probabilities of each word occurrence in the text, weighted by the logarithm of the inverse of these probabilities. The Shannon entropy *H* for a set of words with probabilities *p*_1_*, p*_2_*, ..., p_n_* is calculated as follows:

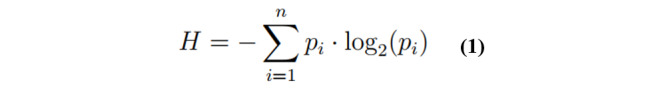



In this equation, *H* represents the calculated entropy value for the given set of words, and *p_i_* represents the probability of the *i*th word occurring in the text. The sum extends over all unique words. By computing Shannon entropy in text analysis, one can gain insights into the richness, diversity, and complexity of the language used within the text dataset.

### Datasets

In this section, we review the specifications of the datasets assessed in this work.

#### University of Maryland Reddit Suicidality Dataset

We used the University of Maryland Reddit Suicidality Dataset (UMD) [93,94] collected from Reddit. Reddit is an online website and forum for anonymous discussion on a wide variety of topics. The UMD is a collection of Reddit posts and comments created by individuals who expressed suicidal thoughts or behaviors. The dataset contains over 100,000 posts and comments collected from various subreddits, including those related to mental health and suicide prevention, such as *Depression* [95] and *SucideWatch* [96] subreddits. The data were collected over a period of several years and include the content of posts and comments, as well as the location and timing of the posts. This dataset contains annotations at the user level, using a 4-point scale to indicate the severity of suicide risk: (1) *no risk*, (2) *low risk*, (3) *moderate risk*, and (4) *high risk*. The goal of this annotation is to assess the risk level of individuals through an examination of their online activities. This task necessitates minimal data, with users generally contributing only a limited number of posts on SuicideWatch. Among the 993 labeled users, 496 made at least one post on the SuicideWatch subreddit. The remaining 497 users served as control subjects. Since the provided labels were user-level labels, we aggregated all the posts of each user into a single data point, through the concatenation of all the posts made by a particular user. Using a binary classification approach similar to that by Ghanadian et al [26], we applied binarization to the UMD. In accordance with the 4 class definitions, the categories labeled as “*no risk*” and “*low risk*” were categorized as nonsuicidal, while those labeled as “*moderate risk*” and “*high risk*” were considered suicidal. A total of 490 anonymous posts with binary labels were employed in this paper.

The UMD has been repeatedly used by researchers to develop and test NLP algorithms and machine learning models in order to identify and analyze patterns in online communication related to suicide risk [97]. Ji et al [98] proposed a method for improving text representation by incorporating sentiment scores based on lexicon analysis and latent topics. Additionally, they introduced the use of relation networks for the detection of suicidal ideation and mental disorders, leveraging relevant risk indicators. In another paper, Ji et al [99] used 2 pretrained masked language models, MentalBERT and MentalRoBERTa, specifically designed to support machine learning in the mental health care research field. The authors assessed these domain-specific models along with various pretrained language models on multiple mental disorder detection benchmarks. The results showed that using language representations pretrained in the mental health domain can enhance the performance of mental health detection tasks, highlighting the potential benefits of these models for the mental health care research community.

#### Knowledge Aware Assessment Dataset

Gaur et al [100] developed an annotated gold standard dataset of 500 Reddit users out of 2181 potentially suicidal users, using their content from mental health–related subreddits within the time frame of 2005 to 2016. The dataset consists of 5 different categories of suicidality, including suicidal ideation, suicidal behavior, actual attempt, suicide indicator, and supportive.

Suicidal ideation refers to thoughts of suicide, which may involve concerns related to suicide risk factors, such as job loss or the end of a significant relationship. Suicidal behavior is defined as actions that carry a higher risk, such as self-harm (either current or historical), active planning to commit suicide, or a history of institutionalization for mental health reasons. An actual attempt encompasses any deliberate action that could potentially lead to intentional death. This includes, but is not limited to, instances where an individual sought help, reconsidered their decision, or publicly expressed thoughts of suicide. The suicide indicator category serves as a classification method to distinguish individuals who use at-risk language from those who are actively experiencing general or acute symptoms. Often, users converse in supportive conversations and share their personal histories while using language from the clinical lexicon. The supportive category pertains to individuals engaging in discussions without expressing any history of being at risk, either in the past or at present.

#### 2021 Reddit Dataset

In addition to the existing datasets, we collected a new dataset of suicidal social media posts from the Reddit platform using the Reddit application programming interface [101]. Specifically, we focused on the “SuicideWatch” subreddit to gather posts related to suicide and analyzed the topics discussed within this subreddit.

The initial data collection was conducted on September 11, 2021, with the goal of gathering 2500 posts published between May 1, 2021, and September 1, 2021. Subsequently, we conducted extensive text preprocessing, which included removing links, eliminating duplicates, handling special characters, removing stop words, filtering out irrelevant and noninformative posts, performing lemmatization, and conducting spellchecking. After these preprocessing steps, our dataset consisted of 2052 unlabeled posts from the “SuicideWatch” subreddit, which we used for the purpose of topic modeling in this study.

#### Synthetic Dataset

Ghanadian et al [26] generated a synthetic dataset to enhance the performance of state-of-the-art suicide detection approaches by augmenting real-world datasets. They harnessed the capabilities of 3 generative large language models, namely, GPT-3.5 Turbo, Flan-T5, and Llama 2, to generate synthetic data for the detection of suicidal ideation.

In this paper, we generated a dataset comprising 748 suicide-related posts. As discussed in the Social and Psychological Knowledge Extraction section, our updated suicidal risk factors and topics, derived from an exhaustive literature search, have led to an extension of the synthetic dataset initially created by Ghanadian et al [26]. We found 5 more risk factors associated with suicidal ideation in the literature. We prompted GPT-3.5 Turbo to generate both suicidal and nonsuicidal instances to add to the existing dataset. This dataset is binary, categorized into suicidal and nonsuicidal classes, with annotations independently provided by 2 expert human annotators. A notable 90% of the labels, initially generated by GPT-3.5, were agreed upon by the human annotators. However, for the remaining 10% of the data, the labels were altered based on the decision of the annotators. In cases where both annotators agreed on a label, that label was retained. Conversely, when disagreements arose, the annotators engaged in discussions to ultimately reach a consensus on the appropriate label.

#### Augmented Dataset

Data augmentation involves enriching a dataset by introducing variations to its existing instances or generating entirely new instances. This process is designed to enhance the diversity and quality of the dataset, which, in turn, can lead to improved model performance and generalization. Ghanadian et al [26] reported that when synthetic data were augmented with 30% of the UMD training set, the fine-tuned model outperformed the model trained with the full UMD. This ratio represents the minimum amount of augmentation required for the ALBERT model to surpass the performance results of the model trained solely on the UMD. Hence, we augmented 30% of the synthetic dataset with the UMD to achieve a balance between diversity and quality in the training dataset.

The augmented dataset was used to fine-tune the pretrained state-of-the-art models and then was evaluated on 2 separate testing sets. The first testing set was 20% of the UMD before any training. The second testing set was generated and annotated by Ghanadian et al [26]. They used several synthetic datasets to create the testing set for their evaluation.

### Ethical Considerations

This study involved the analysis of suicidal posts from social media, which is a sensitive and ethically complex task. We obtained ethics approval for the secondary use of data from the Research Ethics Board at the University of Ottawa (approval number: H-02-23-8967). This approval confirms that our research complies with the ethical guidelines for working with human-derived data and protects the identity of individuals whose posts are analyzed. The data used in this study are from the UMD, which was accessed with authorization from its creators. This dataset was approved by the University of Maryland’s Institutional Review Board. The dataset consists of publicly available social media posts collected from Reddit, where users are anonymous by default. To further protect user privacy, Reddit usernames in the dataset have been replaced with numeric identifiers [102]. Since this study involves the secondary analysis of publicly available and anonymized social media data, informed consent from individual users was not required.

## Results

### Overview

**Figure 3:**
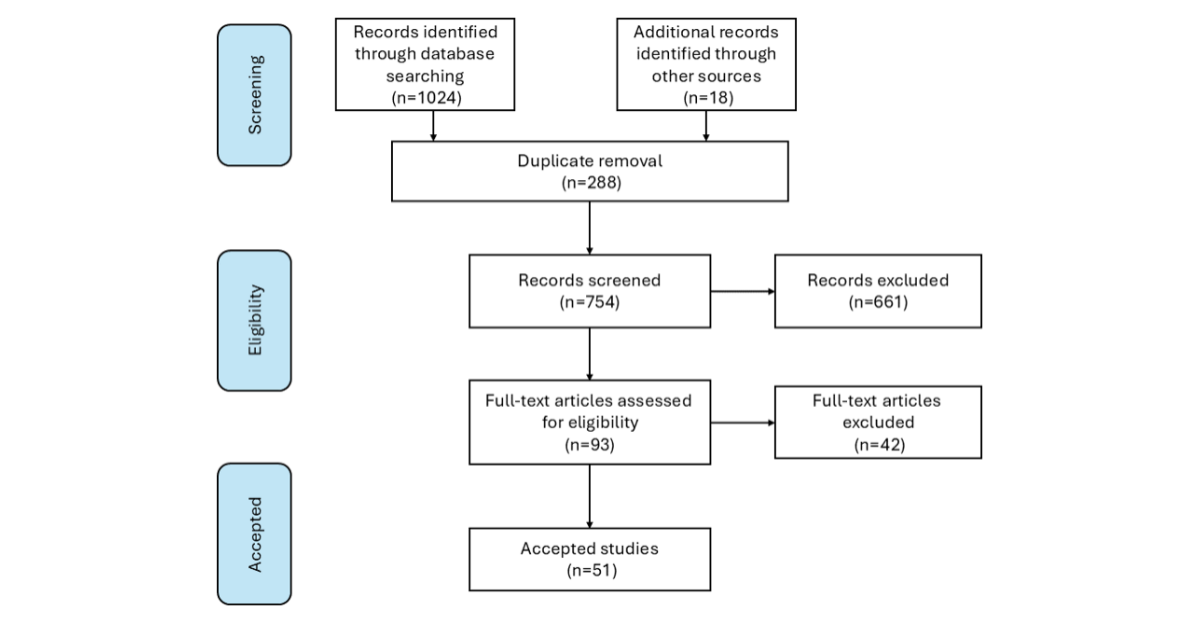
Scoping review workflow for extracting suicide risk factors from psychology literature.

We present the results according to this project’s main research questions. First, we present the discovered topics in 3 real datasets collected from social media, including the UMD, Knowledge Aware Assessment dataset, and 2021 Reddit dataset uncovered by unsupervised BERTopic. Second, we present the results of a scoping review and the reference table for each risk factor. Figure 3 presents the details of our scoping review process. Third, we present the results of topic discovery using guided BERTopic and a comparison between unsupervised and guided BERTopic. Finally, we provide an insightful breakdown, demonstrating the distribution of each topic within our synthetic dataset and the augmented dataset.

To delve deeper into our investigation, we report the performance of the fine-tuned model on these synthetic and augmented datasets, offering a comprehensive comparison between the topic-diverse synthetic dataset and real dataset.

### Unsupervised Topic Modeling

In this section, we assess the topic diversity in all datasets using basic BERTopic. Table 2 reports the most occurring topics and keywords in the selected datasets using basic BERTopic. Some topics are common across all 3 datasets, while others are not discussed. For example, the topic of *education pressure* is discussed in the UMD and 2021 SuicideWatch datasets but is not found in the Knowledge Aware Assessment dataset. The output of topic modeling consisted of clusters of keywords, each associated with a probability of their relevance to the cluster. The title of each topic was chosen based on the highest probability keywords within the cluster.

**Table 2:** Distribution of suicide-related psychological and social risk factors identified through unsupervised BERTopic modeling in Reddit datasets.

Topics and risk factors extracted from psychology			Dataset				
			UMD^a^ (SW^b^ subreddit) (n=490), n		Knowledge Aware Assessment dataset (n=500), n		2021 SW dataset (n=2050), n
**Mental health disorders**							
	Depression	98		78		756	
	Anxiety	45		73		244	
	PTSD^c^	0		0		4	
**Sociodemographic status**							
	Unemployment	18		5		0	
	Financial crisis	0		21		0	
	Education pressure	15		0		28	
**Abuse**							
	Being bullied	5		0		6	
**Family domain**							
	Family issues	50		14		34	
	Relationship problems	17		21		30	
**Personality and psychological traits**							
	Hopelessness	9		0		8	
	Racism	0		0		17	
	Substance abuse	4		0		0	
	Chronic physical problems	0		1		3	

^a^UMD: University of Maryland Reddit Suicidality Dataset.

^b^SW: SuicideWatch.

^c^PTSD: posttraumatic stress disorder.

### Scoping Review Knowledge Extraction

In our scoping review of psychology literature, we initially identified 1042 articles related to suicide. After applying the inclusion and exclusion criteria outlined in Figure 3, a total of 51 studies were retained for analysis. Table 3 provides a list of references we investigated to extract underlying risk factors of suicidal ideation. This table organizes all the studies reporting the identified risk factors. Some of these risk factors fall under broader categories, allowing for a more structured understanding. In the Social and Psychological Knowledge Extraction section, we have provided a description of each extracted risk factor and how it plays a role in suicidal ideation.

**Table 3:** List of risk factors for suicidal ideation extracted through a scoping review of 51 psychology and mental health studies.

Category		Studies
**Mental health disorders**		
	Depression	[12,41-43,103-111]
	Anxiety	[44,106-108,110,112-114]
	Bipolar	[12,17,103,109-111]
	Schizophrenia	[12,103,105,107,109,110,113]
	Borderline	[12,106,110,115,116]
	PTSD^a^	[45,46,107]
**Sociodemographic status**		
	Unemployment	[12,55,107-109]
	Education pressure	[61,62,109,111]
	Financial crisis	[44,60,107,108,111,114]
	Sexual minority stigma	[63-67,107,111]
**Abuse**		
	Being bullied	[44,68-70]
	Sexual abuse	[107,109,111,117,118]
**Family domain**		
	Death of loved ones	[74,108,109]
	Family conflicts	[43,55,110]
	Relationship problems	[55,109,110]
**Personality and psychological traits**		
	Hopelessness	[12,41,42,55,108,119]
	Anger	[41,54,107,108]
	Perfectionism	[41,107,109]
	Chronic physical pain	[83-85]
	Dementia	[80-82]
	Racism	[75-77]
	Immigration	[12,78,79,109,111]
	Substance abuse	[56-59,110,111,114]

^a^PTSD: posttraumatic stress disorder.

### Guided Topic Modeling

In unsupervised topic modeling, we investigated the underlying topics within each social media dataset. A comparison between the extracted topics in the Unsupervised Topic Modeling and Scoping Review Knowledge Extraction sections revealed that many topics reported in psychology literature were not discovered in social media using unsupervised BERTopic. The absence of these topics was evident only when compared to the risk factors in psychology literature, and without this comparison, these important gaps in the discussion might have been overlooked.

To address this, we employed guided topic modeling, which focuses on specific risk factors of interest. This approach ensures that important but less frequently mentioned topics are identified and included in the analysis. Hence, the topics from psychology were used as seed topics for the discovery of the topics in these datasets. Table 4 presents the list of seed words used in guided topic modeling to extract topics from the UMD. We conducted seed topic extractions separately for each of the 3 social media datasets. It is important to note that this list of words does not need to be exhaustive and only serves as a hint about the topic for the topic modeling algorithm.

**Table 4:** List of manually curated seed words representing 23 risk factor categories used to guide topic modeling of suicide-related Reddit posts, grounded in terms extracted from psychology studies and verified using the Merriam-Webster Dictionary.

Category	Seed words
Depression	Depressed, sadness, and mentally ill
Anxiety	Anxious, phobia, and stress
Schizophrenia	Delusion
Bipolar	Manic and temperamental
Borderline	Petulant and impulsive
PTSD^a^	War, memory, and accident
Sexual abuse	Assault and rape
Being bullied	Bullying, oppress, and alone
Family issues	Family, parents, mom, and dad
Relationship problems	Wife, husband, partner, girlfriend, and boyfriend
Death of loved ones	Loss, grief, and mourn
Anger	Outrage and annoyed
Perfectionism	Perfection and expectations
Hopelessness	Despair
Unemployment	Job, work, and poverty
Financial crisis	Money and income
Education pressure	College, school, and overwhelmed
Sexual minority stigma	LGBTQ+^b^ and identity
Racism	Discrimination, justice, bias, and hate
Substance abuse	Alcohol, drug, and opioid
Chronic physical pain	Constant, hurt, and escape
Immigration	Moving, loneliness, and culture
Dementia	Alzheimer

^a^PTSD: posttraumatic stress disorder.

^b^LGBTQ+: lesbian, gay, bisexual, transgender, queer.

#### Real Dataset Topic Verification

Here, we aim to understand the distribution of topics in real data using guided topic modeling. Three real datasets collected from social media were investigated for suicide-related risk factors and topics. Table 5 presents the suicide topics in each real dataset along with the number of posts for each topic. Since we were interested in the distribution of these topics in relation to suicidal thoughts in labeled datasets (UMD and Knowledge Aware suicidality), we only considered the suicidal classes for topic modeling and evaluation. The results in Table 5 show that while social media provides a vast and dynamic platform for individuals to express their thoughts and experiences, it may not always comprehensively reflect the nuanced and scientifically established suicide-related topics discussed in academic psychology literature. The conversational nature of social media often includes a wide range of personal narratives, opinions, and language that may or may not align with the structured and research-driven topics found in psychology literature.

**Table 5:** Distribution of suicidal risk factors extracted using guided BERTopic in real datasets.

Topics and risk factors extracted from psychology			Dataset				
			UMD^a^ (SW^b^ subreddit) (n=490), n		Knowledge Aware Assessment dataset (n=500), n		2021 SW dataset (n=2050), n
**Mental health disorders**							
	Depression	115		79		806	
	Anxiety	52		80		297	
	Bipolar	5		2		2	
	Schizophrenia	0		0		0	
	Borderline	0		2		0	
	PTSD^c^	5		0		4	
**Sociodemographic status**							
	Unemployment	30		25		0	
	Financial crisis	0		30		0	
	Education pressure	30		0		33	
	Sexual minority stigma	0		0		0	
**Abuse**							
	Being bullied	8		0		13	
	Sexual abuse	2		0		7	
**Family domain**							
	Death of loved ones	0		0		0	
	Family issues	55		14		55	
	Relationship problems	25		26		38	
**Personality and psychological traits**							
	Anger	0		0		0	
	Perfectionism	0		0		0	
	Hopelessness	9		0		17	
	Racism	0		0		36	
	Substance abuse	7		0		0	
	Immigration	0		0		0	
	Chronic physical problems	0		2		3	
	Dementia	0		0		0	

^a^UMD: University of Maryland Reddit Suicidality Dataset.

^b^SW: SuicideWatch.

^c^PTSD: posttraumatic stress disorder.

Although social media can offer valuable insights into real-world expressions of mental health concerns, researchers need to carefully interpret and validate social media data to ensure their reliability and relevance to the broader body of psychology literature on suicide. Underrepresentation of certain topics in social media data, specifically stigmatized topics, such as conversations around sexual minorities and racism, can lead to models that underperform in cases where users break the stigma and talk about these issues.

#### Topic Verification on Synthetic and Augmented Datasets

We employed guided topic modeling to verify if this method reveals the suicide topics and risk factors in the synthetic and augmented datasets. Note that these topics were used to generate the synthetic data. However, the number of topics discovered by the guided topic modeling did not sum up to the total number of posts, which was 908 for the synthetic dataset and 1055 for the augmented dataset. This discrepancy arose because some posts were associated with multiple topics and represented more than one risk factor. Table 6 displays the suicide topics within each synthetic and augmented dataset, along with the respective number of posts for each topic. We observed that topic modeling showed a relatively equal distribution of topics in the synthetic dataset. Table 6 illustrates how effectively guided BERTopic extracted the topics within the documents. Furthermore, these results demonstrate the significance of the generation of topic-diverse synthetic and augmented datasets for various research and applications within the domain of suicide-related studies.

**Table 6:** Topic coverage in synthetic and augmented suicidal ideation datasets using guided BERTopic.

Topics and risk factors extracted from psychology		Dataset	
		Synthetic dataset (n=908), n	Augmented dataset (n=1055), n
**Mental health disorders**			
	Depression	62	173
	Anxiety	46	68
	Bipolar	19	20
	Schizophrenia	23	23
	Borderline	23	23
	PTSD^a^	22	21
**Sociodemographic status**			
	Unemployment	20	35
	Financial crisis	21	39
	Education pressure	21	20
	Sexual minority stigma	41	42
**Abuse**			
	Being bullied	39	39
	Sexual abuse	18	20
**Family domain**			
	Death of loved ones	40	41
	Family issues	19	20
	Relationship problems	40	43
**Personality and psychological traits**			
	Anger	39	38
	Perfectionism	48	48
	Hopelessness	36	44
	Racism	39	39
	Immigration	20	18
	Substance abuse	40	46
	Chronic physical problems	56	61
	Dementia	39	48

^a^PTSD: posttraumatic stress disorder.

### Dataset Analysis and Evaluation

In this subsection, we first analyze the quality of each dataset based on 3 indicators introduced in the Dataset Analysis section. The comparison between synthetic and nonsynthetic datasets (UMD, Knowledge Aware, and 2021 Reddit) presented in Table 7 reveals distinct patterns in terms of complexity, readability, and Shannon entropy. The synthetic dataset and augmented dataset exhibited relatively high complexity values of 72.77 and 70.27, respectively, which were comparable to that of the 2021 Reddit dataset (72.87) but surpassed those of the UMD and Knowledge Aware dataset, indicating that synthetic data are linguistically or structurally more complex. In terms of content diversity, measured by Shannon entropy, all datasets showed similar means, but the synthetic dataset was significantly more uniform, with very low deviation (*σ*=0.05), compared to the nonsynthetic datasets. Based on the results presented in Table 7, synthetic datasets tend to be more complex and are more controlled and consistent, whereas nonsynthetic datasets provide a broader range of readability and content diversity.

**Table 7:** Linguistic characteristics of real, synthetic, and augmented datasets for suicidal ideation detection.

Dataset	Complexity (type-token ratio)		Readability (Flesch score)		Shannon entropy	
	*µ*	*σ*	*µ*	*σ*	*µ*	*σ*
UMD^a^	61.18	16.15	82.49	10.87	4.31	0.17
Knowledge Aware dataset	49.04	19.53	82.51	15.27	4.17	0.75
2021 Reddit dataset	72.87	20.13	75.76	48.02	4.27	0.30
Synthetic dataset	72.69	18.40	75.80	10.34	4.27	0.05
Augmented dataset	70.26	17.18	77.38	10.80	4.28	0.05

^a^UMD: University of Maryland Reddit Suicidality Dataset.

Moreover, Table 8 indicates that the readability of the synthetic and augmented datasets was more challenging than that of the real datasets. Specifically, 36% of the synthetic dataset fell into the “easy” and “very easy” categories, while 67% of the UMD fell into these categories. In contrast, 33% of the UMD and 64% of the synthetic dataset fell into the “fairly easy,” “standard,” “fairly difficult,” and “difficult” categories. Furthermore, we conducted a statistical *t* test with a significance level of .05 on the readability, complexity, and entropy metrics, comparing the synthetic dataset with each of the real datasets. Our findings indicated a significant difference in Shannon entropy between the synthetic and real datasets. However, no significant differences were observed in terms of readability and complexity between the real and synthetic datasets.

**Table 8:** Distribution of Flesch reading ease scores across all datasets.

Flesch reading ease interval	UMD^a^, n	Knowledge Aware dataset, n	2021 Reddit dataset, n	Synthetic dataset, n	Augmented dataset, n
Very confusing	2	0	10	0	0
Difficult	1	3	30	0	5
Fairly difficult	5	5	50	50	50
Standard	30	25	200	130	140
Fairly easy	100	140	400	270	300
Easy	200	240	600	170	270
Very easy	100	50	350	80	120

^a^UMD: University of Maryland Reddit Suicidality Dataset.

Additionally, we created a binary classifier for detecting suicidal ideation using different datasets. For that, we fine-tuned ALBERT [120], a transformer-based model optimized for performance and speed, using the UMD and our synthetic and augmented datasets, to evaluate the effect of topics and risk factor inclusion on the performance of a pretrained model. To fine-tune these models, we used the Huggingface library [121]. The Huggingface library is an open-source library and data science platform that provides tools to build, train, and deploy machine learning models. We compared our classification results with a baseline ALBERT model fine-tuned on the UMD by Ghanadian et al [10]. We used the Trainer [122] class from Huggingface transformers for feature-complete training in PyTorch. The hyperparameters were selected based on the default values commonly used in similar studies. The final hyperparameters used in our experiments were as follows: learning rate, 2*e^−^*^5^; batch size, 4; dropout rate, 0.1; and maximum sequence length, 512.

Table 9 presents the performance results of fine-tuning the ALBERT model using the synthetic and augmented training datasets. The model fine-tuned on the augmented dataset outperformed the other 2 models, achieving *F*_1_-scores of 0.91 and 0.90 on the UMD and synthetic testing subsets, respectively. Achieving a high *F*_1_-score is important when evaluating classifiers, especially with imbalanced datasets. A high *F*_1_-score indicates that the model is both precise and effective at identifying at-risk cases. This is critical in sensitive domains like mental health, where failing to detect or incorrectly identifying individuals at risk can have serious real-world consequences.

**Table 9:** Performance metrics of suicidal ideation classifiers trained on real, synthetic, and augmented data.

Test set metrics		Nonsynthetic	Synthetic	
		UMD^a^ dataset	ChatGPT dataset	Augmented dataset
**UMD test subset**				
	Accuracy	0.87	0.72	0.88
	*F*_1_-score	0.87	0.81	0.91
**Synthetic test subset**				
	Accuracy	0.67	0.87	0.90
	*F*_1_-score	0.70	0.86	0.90

^a^UMD: University of Maryland Reddit Suicidality Dataset.

## Discussion

This study investigated whether social media data adequately capture empirically established risk factors associated with suicidal ideation and whether synthetic data augmentation can improve the representation of underrepresented risk factors in suicide detection models. Our findings reveal that real-world social media datasets lack coverage of several critical risk factors, particularly those related to marginalized communities, financial crises, racism, and chronic health conditions. By incorporating insights from psychology literature and leveraging guided topic modeling, we identified these gaps and generated synthetic data to enhance topic diversity. Our results demonstrate that augmenting real-world datasets with topic-diverse synthetic data improves the performance of machine learning models in detecting suicidal ideation, as evidenced by an increase in the *F*_1_-score from 0.87 to 0.91 on the UMD test subset and from 0.70 to 0.90 on the synthetic test subset. These findings support our hypothesis that social media data alone do not fully represent the nuances of suicide risk factors and that synthetic data augmentation can lead to more accurate and fair suicidal ideation detection models.

Traditional data collection methods, specifically self-reports, are subject to various kinds of biases, including social desirability, recall, and self-perception biases. While the use of naturalistic data collected from social media mitigates some of the self-report biases and incorporates contextual information, it, unfortunately, introduces other types of biases, mainly selection bias, platform-induced behavior, and public image curation bias. Many demographics are underrepresented on social media, and even if present, they might shy away from expressing their real self. On the other hand, traditional methods are more controlled, and as we have shown in our literature review, larger numbers of topics and demographics have been covered under those studies. For that reason, we compared the social media datasets to risk factors collected in traditional studies to identify underrepresented risk factors.

This study assessed real-world datasets from social media for their coverage of suicidal ideation topics and risk factors. Additionally, it reviewed psychology literature to identify expert-validated risk factors, suicidal language, and terminology. Integrating this knowledge into NLP models can enhance their contextual awareness in identifying and understanding suicidal ideation in social media posts. Leveraging insights from psychology also supports the development of ethically responsible artificial intelligence systems for addressing sensitive mental health issues.

Unsupervised topic modeling and a scoping review of psychology literature provided 2 sets of suicide topics and risk factors. Table 2 summarizes 13 suicide topics identified across 3 social media datasets, while Table 3 lists 23 risk factors extracted from the literature review. These findings offer valuable insights into the context of suicidal ideation. However, the comparison reveals a significant limitation in social media datasets, which is the absence of critical topics essential for addressing suicidal ideation. This gap could create a barrier to the development of robust NLP models for analyzing suicide-related content.

To address the limitations of unsupervised topic modeling, given the risk factors discovered in our scoping review, we performed guided topic modeling on 3 social media datasets (Table 5) for providing a more comprehensive overview of the suicide topics and risk factors present in the data. The results indicate that certain topics missed during unsupervised topic modeling were identified through guided topic modeling. This issue arises when less frequently discussed topics are overshadowed by more dominant ones, causing them to be overlooked by unsupervised methods. In contrast, guided topic modeling can specifically target and extract these less prominent yet critical topics.

Furthermore, we observed that there was a discrepancy in the distribution of topic representations across the datasets. As an example, let us consider the UMD in Table 5, where the topic “anxiety” appears 10.4 times more than the topic “PTSD.” Such disparities in topic representations could lead to an inherent bias within the models. Biases can occur when certain topics are overrepresented or underrepresented. These biases will potentially impact the effectiveness and fairness of NLP models, as they may disproportionately emphasize or neglect certain aspects of suicidal ideation. Therefore, addressing these data limitations and imbalances is crucial to ensure the development of NLP models that provide accurate and equitable insights into this critical issue.

Moreover, we observed that suicidal narratives related to dementia, sexual minority stigmas, immigration, death of loved ones, perfectionism, and anger were never discussed in these datasets, and topics of being bullied, PTSD, substance abuse, and chronic physical problems were rarely mentioned. Financial crises and racism, which are 2 common and important risk factors of suicide, were only discussed in 1 of the datasets, with a relatively low number of examples. The most represented topics in these datasets were depression and anxiety. This observation is not surprising since many other risk factors lead to mental pressure. In social media conversations, users mostly talk about depression and anxiety, which are surface-level symptoms of what they are experiencing and not the root causes that led to these feelings.

In this study, we harnessed the innovative approach of synthetic dataset creation as a means to enhance the fairness and accuracy of NLP models in the context of detecting suicidal ideation. Using the extracted risk factors from psychology, we constructed a synthetic dataset that comprehensively represents the entire spectrum of risk factors associated with suicidal ideation. To leverage the strengths of both real and synthetic data, we augmented 30% of the UMD by incorporating our synthetic dataset. Table 6 reports the distribution of psychological topics within our synthetic and augmented datasets. We applied guided topic modeling on the synthetic and augmented datasets primarily as a sanity check to show that guided topic modeling performs as expected and, therefore, to analyze the content of datasets according to expected topics. In other words, our goal was to verify whether all relevant topics and risk factors were included in the dataset and could be identified by a cluster- and transformer-based model.

Understanding complexity and readability in synthetic datasets helps in ensuring that the generated text aligns with linguistic patterns observed in real-world data. Moreover, these parameters facilitate an assessment of the synthetic dataset, specifically regarding the incorporation of suitable language complexities. This evaluation allows us to examine whether synthetic data replicates language patterns akin to those found in genuine, human-generated content.

Shannon entropy serves as a quantitative measure of diversity, reflecting the range of vocabulary in a dataset. Our study’s finding of a lower Shannon entropy in synthetic datasets despite higher complexity prompts a nuanced discussion. This discrepancy indicates that, despite intricate language patterns, synthetic datasets might lack the diverse lexical richness found in real datasets. Thus, the relationship between diversity and Shannon entropy suggests that achieving linguistic complexity does not guarantee a broad vocabulary range. Hence, augmenting a real dataset with synthetic data can leverage the advantages of both datasets, incorporating the linguistic complexity characteristic of synthetic datasets and the broad range of diversity inherent in real datasets. Moreover, the relationship between diversity and complexity in datasets reveals a fascinating interplay. While complexity often indicates intricate language structures, the presence of a diverse range of expressions and ideas enhances the overall diversity of the dataset. However, as seen in Table 7, a higher complexity in synthetic datasets does not necessarily translate to a higher diversity, as reflected by a lower Shannon entropy. This suggests that complexity might be influenced more by the intricacy of language patterns than by a broad lexical spectrum.

In order to guarantee the quality of the augmented and synthetic datasets, we fine-tuned the ALBERT model, as a binary classifier, using the aforementioned datasets. Following this fine-tuning, we proceeded to rigorously evaluate the model’s performance on 2 distinct testing subsets. As depicted in Table 9, the results underscore an interesting trend. The model fine-tuned on the UMD exhibited a higher performance when evaluated on an in-domain testing subset but showed a lower performance when tested on an out-of-domain testing subset. A similar pattern emerged when the model was fine-tuned on the ChatGPT synthetic dataset. However, an intriguing twist arose with the augmented dataset. This dataset notably enhanced the model’s performance across both the in-domain and out-of-domain testing subsets. This observation implies that the synergistic inclusion of both real-world and synthetically generated data can yield a more diverse NLP model. This research insight illuminates the potential advantages of a hybrid approach, emphasizing the importance of leveraging the complementary strengths of both real and synthetic datasets to improve the overall performance and adaptability of NLP models in the critical domain of suicidal ideation detection.

Our study has some limitations that should be acknowledged. First, high-quality annotated datasets collected from social media are scarce, so this work exclusively used the Reddit dataset. While valuable information on this topic is shared on other social media platforms, such as X and Facebook, to the best of our knowledge, there is no publicly available large annotated dataset that includes such data. Creating new datasets involves both data collection and a careful annotation process performed by multiple experts, making it an expensive undertaking. Second, due to the limitation of data availability and the fact that the classifier was trained solely on Reddit data, it may not perform as effectively on datasets from other social media platforms. Future work should incorporate data from platforms like X to provide broader insights and improve generalizability.

This study highlights the value of integrating domain knowledge from psychology with computational techniques to improve suicidal ideation detection in social media. By examining 3 real-world datasets, we observed that several well-established risk factors, such as chronic illness, racism, immigration stress, and identity-based stigma, were either absent or rarely discussed. To address these topic gaps, we applied guided topic modeling informed by psychology literature and constructed a synthetic dataset with broader thematic representation. Our evaluations showed that this synthetic data, when combined with real data, enhanced topic diversity and preserved linguistic quality. The classifier fine-tuned on the augmented dataset significantly outperformed models trained solely on real or synthetic data, improving the *F*_1_-score to 0.91 on the UMD and 0.90 on the synthetic test set. These results suggest that synthetic augmentation not only fills in content gaps but also leads to more effective and inclusive detection models.

The findings of this study underscore the importance of integrating psychological insights into NLP models to improve the detection of suicidal ideation on social media. By addressing gaps in topic coverage and leveraging synthetic data augmentation, this work contributes to the development of more robust and ethical artificial intelligence tools for mental health applications. Future research should continue refining these approaches by expanding dataset sources and enhancing model interpretability to ensure that technology is both effective and fair in handling sensitive mental health issues.

## Appendix

Multimedia Appendix 1 PRISMA-ScR (Preferred Reporting Items for Systematic Reviews and Meta-Analyses) checklist.

## Abbreviations

C-TF-IDF: class-based term frequency–inverse document frequency
HDBSCAN: Hierarchical Density-Based Spatial Clustering of Applications with Noise
NLP: natural language processing
PTSD: posttraumatic stress disorder
TF-IDF: term frequency–inverse document frequency
UMAP: Uniform Manifold Approximation and Projection
UMD: University of Maryland Reddit Suicidality Dataset

## Data Availability

The generated synthetic dataset and the code used in this study are publicly available on our GitHub repository [123]. The other datasets used in this research, including the University of Maryland Reddit Suicidality Dataset (UMD), are not publicly available and require permission from the creators of the dataset for access. More information on obtaining the UMD can be found on the UMD website [102]. Researchers interested in accessing the UMD should contact the dataset creators directly for permission and further instructions. By making these resources accessible, we aim to promote transparency, reproducibility, and further research on suicidal ideation detection.
