# Supplementary material for: Improving Suicidal Ideation Detection in Social Media Posts: Topic Modeling and Synthetic Data Augmentation Approach
Source: JMIR Form Res. 2025 Jun 11;9:e63272. doi: 10.2196/63272 (PMC12198699; doi:10.2196/63272)
Supplement: Multimedia Appendix 1 [file formative_v9i1e63272_app1.pdf]

Preferred Reporting Items for Scoping Reviews (PRISMA-ScR) Checklist

SECTION	ITEM	PRISMA-ScR CHECKLIST ITEM	REPORTED ON PAGE #
**ABSTRACT**			
Structured summary	1	Provide a structured summary that includes (as applicable): background, objectives, eligibility criteria, sources of evidence, charting methods, results, and conclusions that relate to the review questions and objectives.	☑
**INTRODUCTION**			
Rationale	2	Describe the rationale for the review in the context of what is already known. Explain why the review questions/objectives lend themselves to a scoping review approach.	☑
Objectives	3	Provide an explicit statement of the questions and objectives being addressed with reference to their key elements (e.g., population or participants, concepts, and context) or other relevant key elements used to conceptualize the review questions and/or objectives.	☑
**METHODS**			
Protocol and registration	4	Indicate whether a review protocol exists; state if and where it can be accessed (e.g., a Web address); and if available, provide registration information, including the registration number.	☑
Eligibility criteria	5	Specify characteristics of the sources of evidence used as eligibility criteria (e.g., years considered, language, and publication status), and provide a rationale.	☑
Information sources*	6	Describe all information sources in the search (e.g., databases with dates of coverage and contact with authors to identify additional sources), as well as the date the most recent search was executed.	**N/A**
Search	7	Present the full electronic search strategy for at least 1 database, including any limits used, such that it could be repeated.	☑
Selection of sources of evidence†	8	State the process for selecting sources of evidence (i.e., screening and eligibility) included in the scoping review.	☑
Data items	9	List and define all variables for which data were sought and any assumptions and simplifications made.	☑
Critical appraisal of individual sources of evidence§	10	If done, provide a rationale for conducting a critical appraisal of included sources of evidence; describe the methods used and how this information was used in any data synthesis (if appropriate).	**N/A**
Synthesis of results	11	Describe the methods of handling and summarizing the data that were charted.	☑
**RESULTS**			
Selection of sources of evidence	12	Give numbers of sources of evidence screened, assessed for eligibility, and included in the review, with reasons for exclusions at each stage, ideally using a flow diagram.	☑

SECTION	ITEM	PRISMA-ScR CHECKLIST ITEM	REPORTED ON PAGE #
Characteristics of sources of evidence	13	For each source of evidence, present characteristics for which data were charted and provide the citations.	☑
Synthesis of results	14	Summarize and/or present the charting results as they relate to the review questions and objectives.	☑
**DISCUSSION**			
Summary of evidence	15	Summarize the main results (including an overview of concepts, themes, and types of evidence available), link to the review questions and objectives, and consider the relevance to key groups.	☑
Limitations	16	Discuss the limitations of the scoping review process.	☑
Conclusions	17	Provide a general interpretation of the results with respect to the review questions and objectives, as well as potential implications and/or next steps.	☑
**FUNDING**			
Funding	18	Describe sources of funding for the included sources of evidence, as well as sources of funding for the scoping review. Describe the role of the funders of the scoping review.	**N/A**
